# Residential overcrowding in relation to children’s health, environment and schooling – a qualitative study

**DOI:** 10.1177/14034948231198285

**Published:** 2023-09-18

**Authors:** Johnny C. Lorentzen, Antonios Georgellis, Maria Albin, Marina Jonsson

**Affiliations:** 1 Centre for Occupational and Environmental Medicine, Region Stockholm, Sweden; 2 Institute of Environmental Medicine, Integrative Toxicology, Karolinska Institutet, Stockholm, Sweden; 3 Institute of Environmental Medicine, Environmental Epidemiology, Karolinska Institutet, Stockholm, Sweden; 4 Institute of Environmental Medicine, Occupational Medicine, Karolinska Institutet, Stockholm, Sweden; 5 Department of Clinical Science and Education, Södersjukhuset, Karolinska Institutet, Stockholm, Sweden

**Keywords:** Child, crowding, built environment, public health, education, social conditions

## Abstract

**Aim:**

To explore how overcrowding affects children’s health, environment and schooling.

**Methods:**

A qualitative study was conducted with individual interviews among 20 participants with occupational experience from overcrowded Stockholm areas but diverse in professions, locations and employers. The interviews were recorded, transcribed and analysed with Systematic Text Condensation.

**Results:**

Almost all participants expressed that overcrowding has a negative impact on children’s health, environment and schooling – based on perceptions of precarious and different living conditions for children in overcrowded areas, for example, substandard homes, vulnerability, stress, exclusion, limited resources, lack of learning opportunities, gender differences, confinement, shame, insecurity, conflicts, risk of criminality, and bodily impact, both physical and psychological.

**Conclusions:**

**Our qualitative evidence suggest that overcrowding has a negative impact on children’s health, environment and schooling**.

## Introduction

Article 25 of The United Nations Universal Declaration of Human Rights states that ‘Everyone has the right to a standard of living adequate for the health and well-being of himself and of his family, including . . . housing’ [1]. In line with this, the *WHO housing and health guidelines* by the World Health Organization (WHO) highlights housing as an important public health issue [2]. The guidelines, based on systematic reviews of the literature up to 2018, warrant actions against crowding in dwellings and list several social and health concerns. Further, studies from Sweden show that atopic burden in overcrowded housing is high in relation to poor housing conditions, particularly in vulnerable populations such as among children and adolescents [3,4]. From a historical perspective, many western countries have solid experience with urbanization that led to crowded city slums that represented various perceived threats to ordered society and population health, and led to the development of, for example, building codes and norms [5–8]. Despite this long history, disentangling the causal chains between adverse outcomes and overcrowding remains a challenge. One difficulty concerns the concept of overcrowding, where countries use different definitions to address the issue for different purposes, such as research, statistical reporting, regulation and administration, for example, for allocating housing and delivering social assistance [7,9]. Further, there is a close connection between overcrowding and ‘social deprivation’ of residents, including poverty, and often with ethnic dimensions, involving migrants, immigrants and indigenous populations [6,7]. These are only some of the complexities; we cover this further in the Discussion section. In Sweden, major actions against crowding were taken already during the 1960s–1970s, when a planning project called the Million Homes Programme led to surplus of spacious and ultramodern housing [10]. Paradoxically, some city districts from this time have recently become severely overcrowded [4,11,12], and buildings have deteriorated over many years.

This raises concern regarding the situation of children, for example, in relation to the United Nations Convention on the Rights of the Child [13], which is law in Sweden and covers all activities that affect children, including education and social services policy [14]. Previous European studies have reported poorer academic results in children from overcrowded homes[15,16], again with complexity regarding factors that could influence outcome [16].

In view of this we identified a lack of information regarding Swedish children’s overall situation in the overcrowded neighbourhoods. We sought to obtain this information from experienced people who, in their different professional roles, meet children and families living in overcrowded conditions. Their combined experiences might provide a broad range of valuable perspectives. Our aim is to shed light on the life situation of overcrowded children by exploring how overcrowding affects children’s health, environment and schooling.

## Methods

### Study design

This study was conducted, as previously described in a Swedish report [17], using qualitative methodology with semi-structured interviews according to Kirsti Malterud’s Systematic Text Condensation (STC) [18,19]: participants (selection); interview questions (interview guide); interview and transcription (data collection); analysis of transcribed interviews (data processing and analysis).

### Participants and settings

Occupations involving contact with children in overcrowded neighbourhoods were identified and categorized according to type of function, for example, medical (see below). Professionals with solid experience in relation to the children’s health, environment or schooling were identified and asked to participate. In the event of a positive response an email with an attached letter described the purpose and method of the study (Supplemental material document 1 online). Few respondents declined the invitation, some of which recommended a colleague instead. During interviews, additional relevant categories of occupations and participants were included. An objective was to cover different residential areas in Stockholm County. A total of 20 professionals were selected for interview (Table I), with the following backgrounds.

**Table I.** Participating professionals with experience of children in overcrowded city districts.

Main employer	Occupation	Other relevant experience
Social services	Social welfare officer	
Police	Police	
Police	Civil investigator	Environmental inspector
Property company (private)	Property manager	Dwelling inspector
Insurance company	Pest sanitizer	
Environmental and health protection (municipal)	Environmental inspector	
Paediatric and adolescent medical clinic	Paediatrician	
Health care centre	Paediatric nurse	
Childcare centre	Nurse	
Elementary school	School nurse	Operations manager (pupils’ health)
Municipality (migrant pupil reception)	School nurse	
Municipality (migrant pupil reception)	School nurse	
Family central (municipal)	Counsellor	Social welfare officer (childcare investigations)
Family central (municipal)	Parent advisor	
Introduction to pre-school (migrants)	Teacher	Family educator, family therapist
Open pre-school	Teacher	
Elementary school, high school	Teacher	
Elementary school	Principal	Teacher
Elementary school	Principal	Teacher, special pedagogue
Elementary school	School counsellor	

Categories of occupations: medical functions, pedagogic functions, social and civil order functions, and residential environment-related functions. Main employer: Government authorities, Region Stock-holm (county), municipalities, and private companies dealing with property management and re-mediation. Residential areas (not shown in Table I, to avoid identification of participant(s): Rinkeby, Husby, Kista, Tensta, Hjulsta, Haninge, Hallunda, Jordbro, Fittja, Alby, Bro, Österåker and Södertälje.

### Interview guide

A semi structured interview guide was designed (by authors M.J. and J.L.) regarding children’s health, indoor environment and schooling. The design of the questions allowed for participants to develop their answers and talk freely and follow-up questions were posed for clarification (Supplemental document 2; the investigators’ pre-understanding within brackets). During interview number three it emerged that gender could be an important perspective, and a question was added if there was a difference between girls and boys.

### Interview and transcription

The interviews were performed at the participants’ workplace between September 2018 and April 2019 by two of the authors, with experience of qualitative research (M.J.) and indoor environment research (J.L.), respectively. Both in the interview situation and in the analysis process, we strove to bracket our preconceptions to minimize the impact of our professional and clinical experiences. The 20 interviews were audio recorded, time ranging from 39 to 87 min, average 59 min. All interviews were transcribed verbatim (by J.L.).

### Analysis of transcribed interviews

The interviews were analysed, in Swedish (by M.J. and J.L.), according to the STC method of Malterud [18,19], and then translated into English. STC is a descriptive approach, which presents participants’ vital experiences and searches for the essence of experienced phenomena. STC includes intersubjectivity, reflexivity and feasibility to reach methodological rigour. The analysis was conducted in four steps, as shown in Table II, which involved both de-contextualization and re-contextualization, thus oscillating between the parts and the whole (Table III). In this STC process (Table II), pieces of data were extracted and closely examined (Table II, steps 1–3) and finally integrated into categories (Table II, step 4). This iterative process alternated between the parts and the whole throughout the analytical process (Table II, steps 1– 4). The categories in step 4 (Table III) represent the headings in the results section.

**Table II.** The analysis process of Systematic Text Condensation (STC).

Step	Analysis process (an iterative process of de-contextualization and re-contextualization)
1	Reading of all transcribed text to get an overall picture of the material and the formation of preliminary themes.
2	All meaning units were encoded after the purpose and themes of the study.
3	The codes were summarized into code groups and then divided into sub-groups.
4	Final analysis summarized the essence of each code group and formed descriptions and concepts of categories and sub-categories that became headings and sub-headings, respectively. After quotes from participants, their occupation is given.

**Table III.** Analysis process from preliminary themes to categories (steps 1 to 4, an iterative process of de-contextualization and re-contextualization).

**Preliminary themes** Reading of all transcribed text to get an overall picture of the material and the formation of preliminary themes.	**Codes** All meaning units were encoded after the purpose and themes of the study.	**Sub-categories/sub-groups** The codes were summarized into code groups and then divided into sub-groups/sub-categories.	**Categories** Final analysis summarized the essence of each code group and formed descriptions and concepts of categories and subcategories that became headings and sub-headings, respectively
Unequal conditions	Many in the same apartment Mattresses Over furnished Bad ventilation	Differences in the indoor environment	Different conditions
	Unusual with an own room and computer Difficulties with the Swedish language Difficult to sleep Difficult to concentrate and learn Difficult to study at home with many siblings	Lack of learning opportunities	
	Normal to live overcrowded here Parallel society Living isolated	Living in alienation	
	Poverty Financial deficiencies Bad health in the family Helping each other Sometimes dysfunctional families Language difficulties due to parents	Guardian’s resources	
	Boys are outside Girls are at home, helping with homework Girls take care of siblings	Differences in gender	
Uncertainty	Fearful Scared Teenage girls most exposed	Insecurity	Living in a vulnerability
	Feeling trapped Afraid of going out Playing difficulties	Confinement	
	Not possible to invite friends Ashamed of the overcrowded home	Shame	
Stress/frustrations	Anxiety Living with unknown people Transmitted responsibility Worry about study/homework	Stress and frustration	Stressful existence
	Quarrels in the family Conflicts on the schoolyard Quarrels among other residents	Conflicts	
Risk situations	Boys meet outdoors Bad outdoor environment	Risk of wrong company	Can lead to risk behaviours
	Risk for recruitment Criminal activities	Risk of criminality	
Health impact	Difficulties concentrating Impact on children’s development Difficulties sleeping Psychosomatic symptoms Asthma exacerbations	Psychological and physical impact	Bodily impact

## Results

Almost all participants expressed that overcrowding had a negative impact on children’s health, schooling and environment. The essence of participants’ perspectives was categorized into ‘Different conditions’, ‘Living in vulnerability’, ‘Stressful existence’, ‘Can lead to risk behaviours’ and ‘Bodily impact’ (Table IV).

**Table IV.** Results in terms of categories and their overall content, and formed sub-categories.

Categories and their overall content	Sub-categories
**Different conditions**	
One participant expresses how overcrowding affects many aspects of children’s living conditions. You don’t have the same conditions as other children in other city districts because, parents have sort of a bad economy, one thing leads to another it becomes like laying a puzzle or like dominoes falling that, well Mom and Dad don’t have the Swedish language, they are not integrated, they may not have steady employment, permanent employment, they depend on financial assistance or, yes you don’t have your own lease, and it starts piling up and then maybe it’s crowded, and then there are effects of that not doing well in school, well then you’re no good at that, I think it’s very important to have a good housing situation. (20, social services) Taken together, the participants express several areas of different conditions.	Differences in the indoor environment
	Lack of learning opportunities
	Living in alienation
	Guardian’s resources
	Differences in gender
**Living in vulnerability**	
Participants describe vulnerability due to feelings of unsafe neighbourhoods and dwelling that are perceived as insecure, being shared with unfamiliar people. That you have mixed families with children, together with addicts, alcohol or drugs, that you have difficulties to maintain your hygiene, because it is like many people share a shower . . . that you may not dare go to the kitchen, you may have sleeping difficulties. (17, principal) As families have no or few alternatives, they may feel trapped and confined, as well of feeling shame of their homes.	Insecurity
	Confinement
	Shame
**Stressful existence**	
Participants describe that overcrowding causes stress in children, for example, relating to pressure on their parents and their own lack of own space to prepare for school. This may lead to conflicts both at home and in school.	Stress and frustrations
	Conflicts
**Can lead to risk behaviours**	
Participants describe that overcrowding can lead to risk behaviours because children with no space at home hang around outdoors and risk contact with criminality and ending up in criminal gangs.	Risk of wrong company
	Risk of criminality
**Bodily impact**	
Participants describe several ways that overcrowding affects the mental state of children, as well as their health and well-being.	Physical and psychological impact

### Different conditions (category)

The participants’ expression of Different conditions (see Table IV) is described below, in the sub-categories Differences in the indoor environment, Lack of learning opportunities, Living in exclusion, Guar-dian’s resources, and Differences in gender.

#### Differences in the indoor environment (sub-category)

The environment of an overcrowded home is different in several ways. Many families rent in second- or third-hand and some pay usury rent. Often, many people live in the same dwelling, for example, a family with relatives, and even several families. Sometimes 10–15 people live in a three-room apartment, each family having one private room and sharing other rooms, for example, bathrooms and kitchens. There may be mattresses all over, the floor flooded with things, making it difficult to keep clean.

It’s full, it’s over furnished, there are many beds and mattresses, often it’s not beds with legs, but mattresses that you can put aside so they have space during the day. (property manager)

Several participants describe substandard ventilation and air inlets sometimes being covered because residents are afraid of draughts and cold. Excess moisture occurs, caused by washing clothes and many taking showers. Some apartments have problems with mould, and many have problems with bed bugs. Many participants express that housing companies have poor control of the apartments, who lives there, wear and tear, and sanitation.

#### Lack of learning opportunities (sub-category)

Participants describe that for schoolchildren in overcrowded dwellings, it is difficult to get peace and quiet when doing homework. Small siblings or other residents may sometimes disturb. For some, it may be difficult to concentrate and take in knowledge, difficult to remember things. Poor sleep contributes to this. Many children must move frequently, changing residence, which can lead to poorer learning.

Yes, then I can’t say 100% that it is due to overcrowding . . . I definitely think that can be a contributing factor, to almost never getting peace and quiet, and if you don’t have the ability to find your own quiet moment, then you have something pushing you all the time, and then this creates anxiety in the body, you have difficulty focusing, to take in longer instructions, briefings, difficulty grasping longer texts, because of that. (school counsellor)

Some children have parents who do not know the Swedish language, and children may not get the help they need for homework. That some children do not master the Swedish language may also affect their learning in school.

. . . and that the parents do not have the ability to help because you maybe don’t have the language. (social services)

#### Living in alienation (sub-category)

Several participants expressed that overcrowded families live as in parallel society, outside the Swedish society. Children often do not go to other parts of Stockholm.

The children have very little knowledge of what society looks like, how ‘Swedes’ are like and live . . . the children have become more isolated out here, so they can have a lot of fantasies about what Swedes are like, because they never meet Swedes, how Swedes live. (teacher)

#### Guardian’s resources (sub-category)

Participants des-cribe that many families in overcrowded neighbourhoods are poor and unable to give the children an own place to sit and study and provide material needs, for example, a computer needed in school. Parents often cannot afford to enter their children in sports and other leisure activities.

Overcrowding is also an effect . . . of the fact that you don’t have the financial resources, and then you become affected. (nurse)

Families are subjected to great stress, partly financial, but if the family is functional, the children may still do well and feel good. The participants also expresses that in these overcrowded areas, many families live with bad health, which in turn can lead to dysfunctional families.

. . . like this, if a family is functional, and you’re feeling good, then overcrowding is not a problem, because then you find solutions, the problem is surely that very many, at least in our catchment area, are not such functional families, this is rather a very vulnerable area. (principal)

Sometimes family relatives also live in the same apartment. It becomes crowded but then there are many adults to help with the children, giving advice and support to the parents. Often, overcrowded children must take responsibility for their family at an early age. They are solidary with their family, seeing their parents having difficulties, mentally, physically and socially. Children often learn Swedish rapidly and then interpret for their parents. Often parents must work hard to pay rent and the children may be left alone at home at an early age. They may be asked to go alone to, for example, a health care centre; young girls may even bring their smaller siblings to medical check-ups.

Children generally have a great solidarity with their parents, but children here maybe even more so, because they have seen their parents having a hard time, and that sometimes, you don’t really count on the parents in this community, it is surely usually the case that children learn the language first, and then they get to interpret for their parents, that is basically really crazy. (teacher)

#### Differences in gender (sub-category)

Participants des-cribe gender differences in the overcrowded areas. Boys are free to hang out late at night. Girls must often be at home after school, taking care of younger siblings, helping with cooking and baking. Several participants experience girls in overcrowded neighbourhoods having better grades than boys.

Well a lot of girls here surely help a lot at home and this is something we might not always be aware of, but the school becomes their free zone in some way, so it’s both an effect of overcrowding but also of a cultural way of thinking, that you’re going home and then you help your mother, especially if you have a lot of younger siblings, and it falls on the girls, there you surely have another thing, that the boys have more freedom. (police)

### Living in vulnerability (category)

The participants’ expression of Living in vulnerability (see Table IV) is described below, in the sub-categories Insecurity, Confinement, and Shame.

#### Insecurity (sub-category)

Participants describe overcrowding as an insecurity for the children and their family because the apartment may sometimes be shared by many people with different cultures and religions, and addicts. Parents may feel insecure and fearful, which may lead to children being scared. Teenage girls are most vulnerable when their residence is shared with non-familiar adult men and teenage boys. No specific undue event is stated, but participants describe teenage girls feeling discomfort that something might happen. This sometimes leads girls to avoid showing themselves. Girls may also feel vulnerable in being girls, perceiving their gender to have lesser value, and their culture; having to wear clothing such as a shawl enhances their vulnerability. Girls may be afraid of moving about outdoors because the neighbourhoods do not feel safe.

#### Confinement (sub-category)

Children become confined as parents feel trapped, being afraid of other people in the apartment and sometimes also of going out. They are not able to play as they like at home and outside.

They were hiding inside this house, they sort of confined themselves, so that there was no free play for the children in any way, they sat silent on the couch when we were there, clinging to Mom and when we tried talking to their mother, we had the property manager with us, she was extremely tense and didn’t want to answer any questions. (civil investigator)

#### Shame (sub-category)

Participants also describe children in shame for not being able to invite friends to their home after school, not wanting to show their overcrowded homes.

. . . because there is no place, then you maybe avoid hanging out with friends because everyone else has a home you can go there and things like that, but you can never invite your friends, so it really has a huge importance this with overcrowding for children and young people. (school nurse)

### Stressful existence (category)

The participants’ expression of a Stressful existence (see Table IV) is described below, in the sub-categories Stress and frustrations and Conflicts.

#### Stress and frustrations (sub-category)

Participants describe that parental stress, due to the housing situation and economy, may be transmitted to their children, which may cause anxiety attacks and aggravate somatic diseases. Children are also stressed by living with people they do not know. Participants also described children stressed by not being able to study before going to school, as they have no adequate place for homework. Taking adult responsibilities also creates stress.

You pick up your younger siblings at preschool or initial classes, you take them home and wait for Mom or Dad or an adult should come home and sort of take over, and then you don’t get peace and focus to concentrate on school work anyway, and also that you become, or can become in my experience, maybe a little messier, loud now I don’t want to say that about pupils, but it becomes an anxiety in the body,. . . (school counsellor)

#### Conflicts (sub-category)

Participants describe conflicts occurring when people are overcrowded, sometimes related to families from different cultures and religions living in the same apartment and sharing spaces. That children do not have space of their own, where they can withdraw, also creates conflicts, for example, between siblings, and causes stress in children. Often occurring conflicts in schoolyards may also partly be due overcrowding at home. Children have no sphere of their own at home, they feel monitored, and therefore become closer to conflicts.

It’s crowded and it gets hot and it’s easy to get conflicts, and the children wake up crying and, the parents often carry substantial trauma. (school nurse)

### Can lead to risk behaviours (category)

The participants expression of Can lead to risk behaviours (see Table IV) is described in the sub-categories Risk of wrong company and Risk of criminality.

#### Risk of wrong company (sub-category)

Participants describe children having no space at home, and they cannot bring their friends. These youths, usually boys, then meet outdoors, close to schools, youth recreation centres or in city district centres, and easily end up in the wrong company.

. . . where the young people hang out, and this is of strategic importance because, you have to go through this area, when you go to and from the youth recreation centre, and we know that this is where recruitment takes place . . . (civil investigator)

#### Risk of criminality (sub-category)

Participants des-cribe that when adolescents get in the wrong company, they risk being picked up by criminal gangs, and may be recruited for criminal acts. It may start with selling cigarettes and other drugs, and small burglaries, which can lead to more serious crime.

. . . and precisely because these crimes are not solved, and these criminal gangs have a strong grip in these areas, I think this is almost the worst right now anyway. (teacher)

### Bodily impact (category)

The participants’ expression of Bodily impact (see Table IV) is described in the sub-category Psychological and physical impact.

#### Psychological and physical impact (sub-category)

Participants describe overcrowded children having more difficulty concentrating and are often louder and rowdier than other children. Overcrowding also leads to children lacking opportunities to reflect on things and situations that happened during the day and, being constantly disturbed, they are unable to develop their own thoughts and identity. This may be especially problematic in adolescence when it is important to develop your own self and become independent.

. . . But I think that as you get older, when you become a teenager, you need your own sphere it is also part of freeing yourself from your parents. (principal)

Children have difficulty sleeping as their homes are constantly noisy in the apartment, and some children may have traumatic experiences from their home country or journey to Sweden. Participants describe children with constant unrest that can manifest in different ways, for example, anxiety, depression and psychosomatic symptoms, for example, headaches and stomach pains. Girls may sometimes become low when not being allowed to go out.

. . . partly this cultural thing, they can’t go out with their friends, then you sit at home, in a home where you also have no space to do what you want, and then they really become low. (school nurse)

Participants describe schoolchildren potentially having difficulty keeping good hygiene, maybe due to lacking access to a bathroom. Children are more likely to suffer from bed bugs, which are common in their overcrowded neighbourhoods. Overcrowding also leads to recurring colds in children. Those with asthma may be impaired by their home environment. Their asthma may also be more difficult to treat.

. . . especially in the winters, cold after cold, infection after infection, because the door is closed, you’re sitting in a room with 12 square metres together with 4–5 other people all the time. (school nurse)

## Discussion

Qualitative studies may be influenced by the investigators’ own world views and professional experiences. Here, we tried to mitigate such bias and limitation by bracketing our preconceptions during both interviews and analysis. Furthermore, we tried to increase the robustness of our results by including many participants with heterogenous backgrounds. While our study cannot be used for generalization, as can quantitative studies, we did capture depth and variation of responses from the different professionals that meet children in overcrowded areas. This gave us a broad perspective on how children are affected in overcrowded homes, that is, precarious and different living circumstances, substandard housing, vulnerability, stress, exclusion, limited resources, lack of learning opportunities, gender differences, confinement, shame, insecurity, conflicts, risk of criminality, and bodily impact, both physical and psychological. We conclude therefore that overcrowding affects children’s health, environment and schooling in many ways. Our results may give professionals who meet these children some basis for the implementation of activities that might reduce the effects of overcrowding on children’s health, environment and school performance.

Almost all participants expressed that overcrowding has a negative impact on children’s health, environment and schooling. This troubling result may be relevant not only for Stockholm, as similar overcrowded districts from the Million Homes Programme occur all over Sweden. Indeed, similar results have been reported from Rosengård in Malmö, for example, severely overcrowded dwellings with mould and vermin [4]. There, the inhabitants were also predominantly of foreign background and their estimated number was close to 8000, whereas the official population was 5000 in an area originally planned for 3000 [4]. Furthermore, the authors state that immigrant populations generally differ from the resident Swedish population in terms of poverty, health outcomes and educational outcomes [4]. Several Swedish Governmental Agencies focus on these overcrowded city districts, for example, social services and the police [20]. In line with this complexity, the participants in our study did not view overcrowding as the only cause of deficiencies, they also mentioned other factors, for example, segregation of urban areas, cultural and ethnic differences, financial constraints, traumatic experiences from home, et cetera. Furthermore, a recent WHO review, *WHO housing and health guidelines*, calls for caution against causal attribution to crowding, due to, for example, the close relationship between crowding and social deprivation [2]. In line with this, a recent review suggests that multiple hazardous dwelling condition characteristics may often coincide in overcrowded dwellings [7]. Thus, multiple environmental factors may be relevant for multiple health outcomes. In addition, behaviours are also important; the WHO recommends further research on intimate partner violence and mental health in relation to crowding, and also calls for public health action [2].

Concerning the indoor environment, some Swedish studies do indicate that shortcomings are common in overcrowded dwellings, including mould and cockroach infestation [3,4]. Our study adds to this that bed bugs appear also to be common. To the best of our knowledge, no systematic scientific studies have investigated the consequences of the increasing overcrowding on indoor environments in Sweden, but again, this is inherently difficult to study. This is true also for the other potential outcomes of overcrowding that we addressed, that is, health and schooling, at least in the city districts we studied, with overcrowded homes and neighbourhoods.

In Sweden, it may take considerable time before the housing stock can meet the demand in line with national codes and norms. Still, some negative consequences of overcrowding can be amended by simple strategies that include information to residents, in their own language when needed. For example, since large family size [21] and overcrowding [4,11,12] can lead to increased humidity, the risk of indoor mould may be reduced by adjusted behaviours, for example, in relation to bathing, cooking, drying laundry, et cetera [22]. Moreover, housing inspections especially focusing on poorly maintained housing stock with vulnerable residents and remediating actions are warranted. Further, on a societal level high priority should be given to efforts to compensate for the lack of residential indoor space in areas with severe overcrowding, for example, to provide a safe, accessible outdoor environment supporting recreation, as well as diverse accessible indoor spaces outside the home, both calm ones for children’s homework and those permitting social engagement and joint activities.

Despite the complexity of studying the effects of overcrowding, it is essential to follow up on our results with relevance for public health of participants expressing that overcrowding has a negative impact on children’s living conditions, health, environment and schooling. This is in line with the Dahlgren–Whitehead rainbow model of important health determinants to promote social equity that is reflected in our results [23]. Everyone who meets and cares for these children, whether working in medical, educational or social functions, needs to identify these children and deploy preventive factors to reduce risks of negative effects of overcrowding, in accordance with the UN Convention on the Rights of the Child [13].

Finally, we learned during interviews that several schools offer various compensatory measures, such as help with homework at school, cultural activities outside their overcrowded neighbourhoods, et cetera. [17]. It should also be pointed out that some participants mentioned circumstances that could favour children in larger and extended families living in the overcrowded homes, for example, more adults and youth to help younger children with homework, et cetera. These leads, and others presented here, could be interesting to follow up, for example, in qualitative studies that obtain the information directly from the children and adults living in overcrowded conditions. This includes their perspective on mitigation measures that would improve the possibility to satisfy the basic physical, mental and social needs of family members of different ages in the overcrowded homes and city districts.

## Supplemental Material

sj-docx-1-sjp-10.1177_14034948231198285 – Supplemental material for Residential overcrowding in relation to children’s health, environment and schooling – a qualitative study Supplemental material, sj-docx-1-sjp-10.1177_14034948231198285 for Residential overcrowding in relation to children’s health, environment and schooling – a qualitative study by Johnny C. Lorentzen, Antonios Georgellis, Maria Albin and Marina Jonsson in Scandinavian Journal of Public Health

sj-docx-2-sjp-10.1177_14034948231198285 – Supplemental material for Residential overcrowding in relation to children’s health, environment and schooling – a qualitative study Supplemental material, sj-docx-2-sjp-10.1177_14034948231198285 for Residential overcrowding in relation to children’s health, environment and schooling – a qualitative study by Johnny C. Lorentzen, Antonios Georgellis, Maria Albin and Marina Jonsson in Scandinavian Journal of Public Health
