# Supplementary material for: Residential overcrowding in relation to children’s health, environment and schooling – a qualitative study
Source: Scand J Public Health. 2023 Sep 18;52(7):829–37. doi: 10.1177/14034948231198285 (PMC11476345; doi:10.1177/14034948231198285)
Supplement: sj-docx-1-sjp-10.1177_14034948231198285 – Supplemental material for Residential overcrowding in relation to children’s health, environment and schooling – a qualitative study [file sj-docx-1-sjp-10.1177_14034948231198285.docx]

**Trångboddhetens effekter på barns hälsa och miljö**

**Välkommen att delta i en intervju om trångboddhet**

Trångboddheten har ökat på senare tid, framförallt i storstäderna. Huvudsakligen är det hushåll med lägre inkomster som bor trängre samt barn, ungdomar och nyanlända och personer med invandrarbakgrund. Idag har vi mycket liten kunskap om hur detta att bo trångt påverkar våra barn och ungdomar eftersom så få studier utförts inom området.

Inom Stockholms Läns Landsting bedriver vi nu ett projekt om trångboddhetens effekter på barns hälsa och miljö där vi intervjuar personer som har kontakt med barn och ungdomar i områden där trångboddhet förekommer.

Vi skulle gärna vilja intervjua dig för att få ta del av dina erfarenheter om hur du upplever att trångboddhet påverkar de barn du möter. Frågorna handlar om hur du upplever att barnen påverkas fysiskt, psykiskt och socialt, samt hur trångboddheten kan påverka deras skolgång, lärande och skolprestationer. Vi kommer även att ställa frågor om barnens bostadsmiljöer, både deras inom- och utomhusmiljö. Då vi kommer att intervjua personer med olika yrkesbakgrund kommer olika frågor ställas vid intervjuerna.

Intervjun tar högst en timme och kommer att spelas in men blir avidentifierad i redovisningen. Därför kommer ingenting du säger kunna kopplas till just dig. Allt material behandlas konfidentiellt. Deltagande är frivilligt och du har rätt att när som helst avbryta intervjun.

Hör gärna av dig vid frågor till någon av kontaktpersonerna nedan:

Med Vänliga Hälsningar

Johnny Lorentzen och Marina Jonsson

*Kontaktpersoner:*

Antonis Georgelis Johnny Lorentzen Marina Jonsson

Enhetschef, docent Miljöhygieniker, docent Allergisamordnare, med dr

Centrum för arbets- och CAMM CAMM

Miljömedicin (CAMM) [Johnny.lorentzen@sll.se](mailto:Johnny.lorentzen@sll.se) [marina.jonsson@sll.se](mailto:marina.jonsson@sll.se)

Tel: 076-7212607 Tel 072-536 5219
