# Supplementary material for: Residential overcrowding in relation to children’s health, environment and schooling – a qualitative study
Source: Scand J Public Health. 2023 Sep 18;52(7):829–37. doi: 10.1177/14034948231198285 (PMC11476345; doi:10.1177/14034948231198285)
Supplement: sj-docx-2-sjp-10.1177_14034948231198285 – Supplemental material for Residential overcrowding in relation to children’s health, environment and schooling – a qualitative study [file sj-docx-2-sjp-10.1177_14034948231198285.docx]

**General questions about the participants´ background**

1. How have you experienced overcrowding? (history: occupations, other activities, for how long)

2. Do you meet overcrowded children or adolescents today? (how, in what contexts, ages)

**Initial question**

3. What are your thoughts regarding overcrowded children and adolescents? (negative/positive)

**Questions regarding impact on health, social and school**

4. How do you perceive that children/adolescents are affected by overcrowding?

Physically (hygiene, body, symptoms, fatigue, infections, diseases, allergies)

Psychological (family situation, stress, mood, fatigue, mobility)

Social impact (conflicts, games, activities, cooperation with other children, adults)

Schoolwork (concentration, learning, homework, school performance)

**Questions about stories**

5. Tell us about any individual case of children and overcrowding that you particularly remember

6. How do children/adolescents talk about overcrowding?

**Questions about the environment**

7. How do you perceive the outdoor environment of overcrowded children? (insecurity)

8. How do you perceive the indoor environment of overcrowded children? (moisture, mould, hygiene, e.g., pests, health)

9. How do you consider that housing and residential areas can be affected by overcrowding? (safety, constructions, ventilation, moisture & mould, hygiene, e.g., pests, health)

**Questions about history, characteristics, and actions**

10. How do you perceive distribution and extent of overcrowding over time? (where is it, increased/decreased, factors that influence, socio-economy)

11. How to reduce the effects of overcrowding on children/adolescents?

12. What do you think about the concept of overcrowding? (definition, confounding)

13. Can you give any examples of important texts on overcrowding?
